# Multicentre, phase II study of eribulin in combination with S-1 in patients with advanced breast cancer

**DOI:** 10.1186/s12885-019-6200-5

**Published:** 2019-10-16

**Authors:** Tsutomu Iwasa, Junji Tsurutani, Satomi Watanabe, Ryoji Kato, Yutaka Mizuno, Yasuyuki Kojima, Tsutomu Takashima, Nobuki Matsunami, Takashi Morimoto, Jun Yamamura, Shoichiro Ohtani, Yuko Tanabe, Tetsuhiro Yoshinami, Toshimi Takano, Yoshifumi Komoike, Kazuhiko Nakagawa

**Affiliations:** 1 Department of Medical Oncology, Kindai University Faculty of Medicine, Osakasayam, Japan; 2 Advanced Cancer Translational Research Institute, Showa University, 1-5-8 Hatanodai, Shinagawa-ku, Tokyo, 142-8555 Japan; 3 Department of Breast Surgery, Yokkaichi Municipal Hospital, Osakasayam, Japan; 4 Division of Breast and Endocrine Surgery, Department of Surgery, St. Marianna University School of Medicine, Osakasayam, Japan; 5 Department of Surgical Oncology, Osaka City University Graduate School of Medicine, Yokkaichi, Japan; 6 Department of Breast Surgery, Osaka Rosai Hospital, Kawasaki, Japan; 7 Department of Breast Surgery, Yao Municipal Hospital, Osaka, Japan; 8 Department of Surgery, Breast Oncology, Sakai City Medical Center, Sakai, Japan; 9 Department of Breast Surgery, Hiroshima City Hiroshima Citizens Hospital, Sakai, Japan; 10 Department of Medical Oncology, Toranomon Hospital, Sakai, Japan; 11 Department of Breast and Endocrine Surgery, Graduate school of medicine Osaka University, Hiroshima, Japan; 12 Department of Breast and Endocrine Surgery, Kindai University Faculty of Medicine, Tokyo, Japan

**Keywords:** Eribulin, S-1, Phase II study, Breast cancer, TNBC

## Abstract

**Background:**

We previously reported the synergistic effect of S-1 and eribulin in preclinical models. In addition, our phase I study revealed the recommended dose for the phase II study of the combination therapy in advanced breast cancer (ABC) patients pre-treated with anthracycline and taxane. Our current study reports on the efficacy and safety of the combined use of eribulin and S-1 in patients with ABC and poor prognosis.

**Methods:**

Patients with breast cancer who received prior anthracycline- and/or taxane-based therapy were assigned to receive a combination therapy of eribulin (1.4 mg/m^2^ on days 1 and 8, every 21 days) and S-1 (65 mg/m^2^, on days 1 to 14, every 21 days) for advanced/metastatic disease. All patients had at least one clinicopathological factor such as being oestrogen receptor negative, Human Epidermal Growth Factor Receptor 2 **(**HER2) receptor negative, presence of visceral involvement, presence of three or more metastatic sites, or having a disease-free interval shorter than 2 years. The primary endpoint was the independent-reviewer assessed objective response rate (ORR). Secondary endpoints were clinical benefit rate, disease control rate, progression-free survival (PFS), and overall survival (OS).

**Results:**

This study enrolled 33 patients. Confirmed ORR was 33.3% (95% CI: 17.3 to 52.8). Median PFS was 7.5 months (95% CI: 4.0 to 14.3). Median OS time was not reached during the current experimental periods. The most common grade 3/4 adverse event was neutropenia (68.8%).

**Conclusions:**

The combination of eribulin and S-1 is safe and effective for treatment in patients with ABC and poor prognosis.

**Trial registration:**

Current Controlled Trials UMIN000015049, date of registration: September 5th 2014.

## Background

Eribulin, which is a non-taxane microtubule polymerization inhibitor, is an FDA-approved agent with documented clinical efficacy and acceptable toxicity profiles in patients with metastatic breast cancer previously treated with an anthracycline and a taxane [1–3]. This drug was also approved in Japan in 2010. S-1 is an oral fluoropyrimidine capsule formulation that has also been shown to exhibit anti-tumour activity and low gastrointestinal toxicity. It has been approved and widely used in Asian countries, including Japan, and is accepted as the standard care for gastric [4–6], colorectal [7], non-small-cell lung [8], and pancreatic cancers [9].

S-1 has also been recommended as an option for first or later-line treatment of patients with advanced breast cancer (ABC) in Japan [10, 11]. These recommendations are based on phase II studies that showed a response rate of 40.7 and 42.0% as first- or second-line treatments, respectively, and 21.8% as a salvage treatment [12, 13]. Furthermore, a phase III study showed that when used as a first-line treatment, S-1 was not inferior to taxane with respect to overall survival [11]. When taken together, these data suggest that S-1 might be a suitable option that can be used to treat patients with ABC.

The primary goal of treatments for ABC patients is to alleviate symptoms from the disease and prolong survival. Several guidelines, including the ABC3, have proposed using combination chemotherapy (CT) to treat these patients. However, it was pointed out that CT should be reserved for patients with rapid clinical progression, life-threatening visceral metastases, or when there is a need for rapid symptom and/or disease control [14]. The characteristics of patients with ABC and poor prognosis have been previously described [15, 16]. Thus, these clinicopathological features might be useful for helping to define patients who are likely to benefit from intensified treatments such as combination CT.

We previously reported the identification of a synergistic effect ofS-1 and eribulin in preclinical models [17], and recommended using the phase II dose of the combination in the patients with ABC who had been previously treated with anthracycline and taxane [18]. Here, we report results of this phase II study of the administration of eribulin in combination with S-1 in patients with ABC and poor prognosis.

## Methods

### Study design and patients

This multicentre, single arm, phase II trial assessed the objective response rate (ORR) of eribulin in combination with S-1 in patients with ABC. Eligible patients were older than 18 years and had HER2-negative metastatic breast cancer. Patients enrolled in the study had not undergone more than one previous chemotherapy regimens for metastatic disease, and they had received neoadjuvant or adjuvant treatment or treatment for metastatic disease with an anthracycline and/or a taxane. Patients had at least one poor prognostic factor (hormone receptor-negative, presence of visceral involvement, presence of three or more metastatic sites, or a disease-free interval shorter than 2 years). Patients needed to have normal baseline organ and bone marrow functions, and a measurable disease according to the Response Evaluation Criteria in Solid Tumors (RECIST), version 1.1, which was assessed by blinded independent central review. Patients were excluded if they had previously received eribulin or S-1 chemotherapy. We additionally excluded patients with central nervous system metastases, active infection, uncontrolled pleural effusion, ascites, and pericardial effusion.

The study was conducted in accordance with the Declaration of Helsinki (2008). The primary investigators submitted the protocol and informed consent form for approval by the Institutional Review Boards at Kindai University (the approval number 26–143) and each of the participating centres. All patients provided written informed consent for participating in the study and publication of the results before undergoing any study-related procedures.

### Treatments

Patients were intravenously administered eribulin mesylate at a dose of 1.4 mg per square meter of body surface area on days 1 and 8, with administrations repeated every 21 days. S-1 was administered orally on a daily basis at a dose of 65 mg per square meter of body surface area (divided into two doses) for 14 days, and repeated every 21 days. The assigned treatment was continued until disease progression or unacceptable toxic effects occurred.

### Study endpoints

ORR was used as the primary endpoint for the eribulin and S-1 combination therapy, which was assessed by an independent reviewer using RECIST version 1.1. The ORR was defined as the proportion of subjects who achieved a complete response (CR) plus those who achieved a partial response (PR). Secondary endpoints included clinical benefit rate (CBR) (defined as the percentage of patients who achieved CR, PR and stable disease (SD)), disease control rate (DCR) (defined as the percentage of patients who achieved CR, PR and SD), overall survival (OS), progression-free survival (PFS), new metastasis-free survival, duration of response (DOR) and toxicity. For patients with measurable disease, CBR was defined as the CR, PR, or SD for at least 6 months (CR + PR + SD >  6 months), while the DCR was defined as CR, PR, or SD (CR + PR + SD). Toxicity was graded according to NCI Common Toxicity Criteria version 3 and was reported for grade 1 to 5 toxicities, which were considered to be possibly, probably, or definitely treatment related. However, the evaluation of Quality of Life (QOL) was not examined in this study.

### Statistical analysis

Efficacy data were analysed on an intention-to-treat basis, with safety assessed in all patients who received at least one dose of the protocol treatment. The primary analysis of ORR was determined along with the corresponding two-sided, exact binomial 95% confidence intervals (CIs). After assessing the response (expected value 35%), we calculated the sample sizes (32 patients) following Simon’s Two-Stage for Phase II clinical trials with *α* = 5%, *β* = 10% and we defined threshold value for ORR as 10% and performed the binomial test for the following hypothesis (H0: ORR = 0.1, H1: ORR > 0.1). PFS, OS, time to recurrence (TTR), and DOR were calculated using the Kaplan-Meier curves. DCR, CBR, and 95% CIs were also determined. For analysis of the new metastasis-free survival, the cumulative incidence competing risk method was used to estimate the cumulative probabilities of disease progression due to the development of a new lesion or death.

## Results

### Patients

**Fig. 1:**
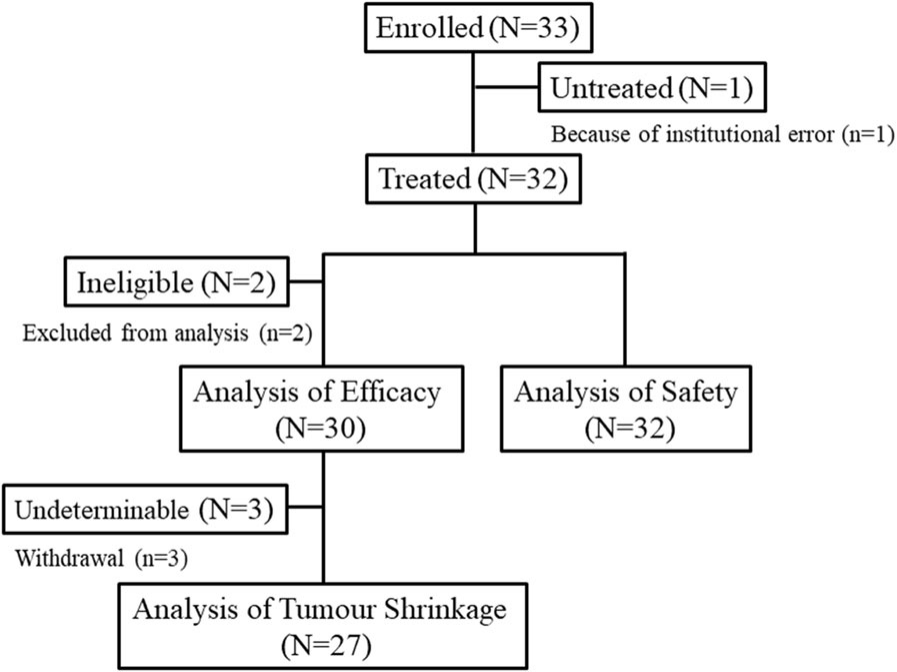
CONSORT Diagram. CONSORT diagram showing details regarding the screening and treatment status. Patients were analysed if they received at least one dose of the experimental therapy. There were 32 patients who received the assigned treatment, of which 30 patients were evaluated for the primary endpoint

A total of 33 patients were enrolled in the study from September 2014 through August 2016. As one patient was not assigned due to an error, there were 32 patients who were assigned a treatment. Of these patients, it was possible to evaluate 30 (two patients did not meet the inclusion criteria) for the primary endpoint (Fig. 1). The median age was 53.6 years, with half of the patients found to have triple-negative breast cancer. Table 1 shows the baseline demographic characteristics. The median follow-up time was 6.8 months.

**Table 1:** Patient demographic and tumour characteristics

		*n* = 32(%)
Age, mean ± SD		53.6 ± 12.2
Hormone receptor	positive	14 (43.8)
	negative	18 (56.2)
TNBC		16 (50)
PS	0	19 (59.4)
	1	13 (40.6)
Prior treatment	Anthracycline	23 (71.9)
	Taxane	29 (90.6)
Prior regimen number	0	11 (34.4)
	1	14 (43.8)
	2	7 (21.9)
Visceral metastasis	present	22 (68.8)
Number of metastases	1	28 (87.5)
	2	2 (6.3)
	3	1 (3.1)
	4	1 (3.1)
Metastatic sites	Liver	14
	Lymph node	10
	Lung	8
	Skin	3
	Pleural	1
	Breast	1
	Thyroid	1
	Spleen	1

*Abbreviations*: *OR* Oestrogen receptor, *PgR* Progesterone receptor, *HER-2* Human epidermal growth factor receptor 2, *PS* Performance status

### Efficacy

**Fig. 2:**
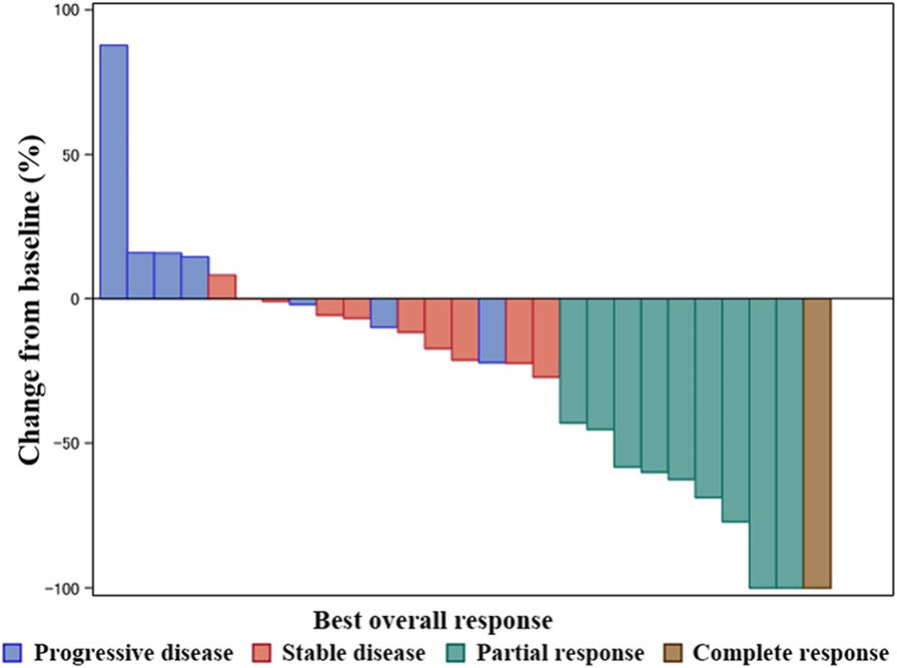
Waterfall Graph of Percentage Change. Waterfall plot analysis showed that most patients (83.3%) exhibited a decrease in tumour size. Waterfall graph of the percentage change in total sum of the target lesion diameters from baseline to post-baseline nadir (RECIST 1.1). Abbreviation: RECIST = Response Evaluation Criteria in Solid Tumours

**Fig. 3:**
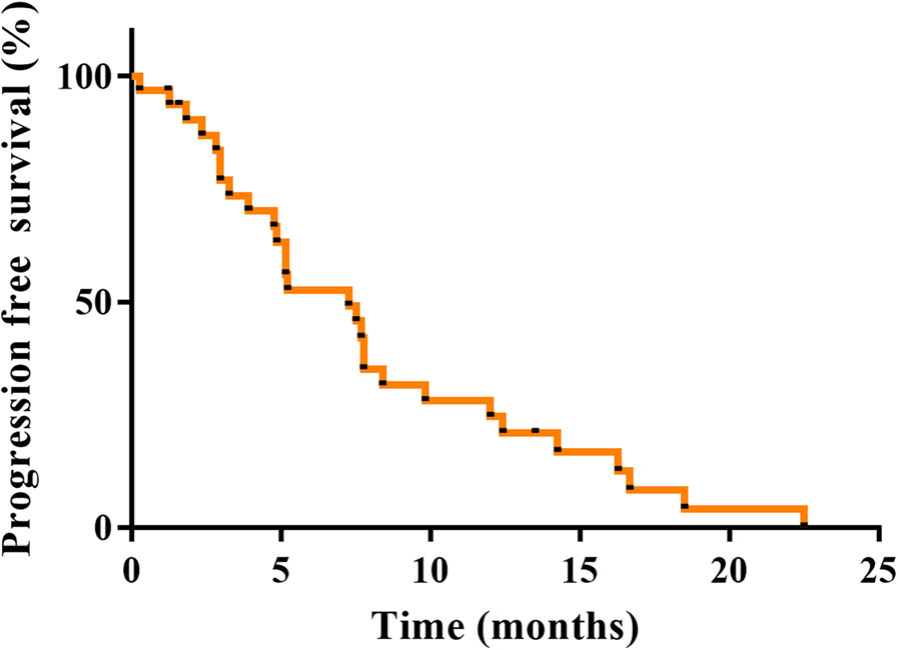
Kaplan-Meier Analysis of Progression-free Survival. The median PFS was 7.5 months (95% CI: 4.0 to 14.3) based on a blinded independent central review. Abbreviation: PFS = progression-free survival

**Fig. 4:**
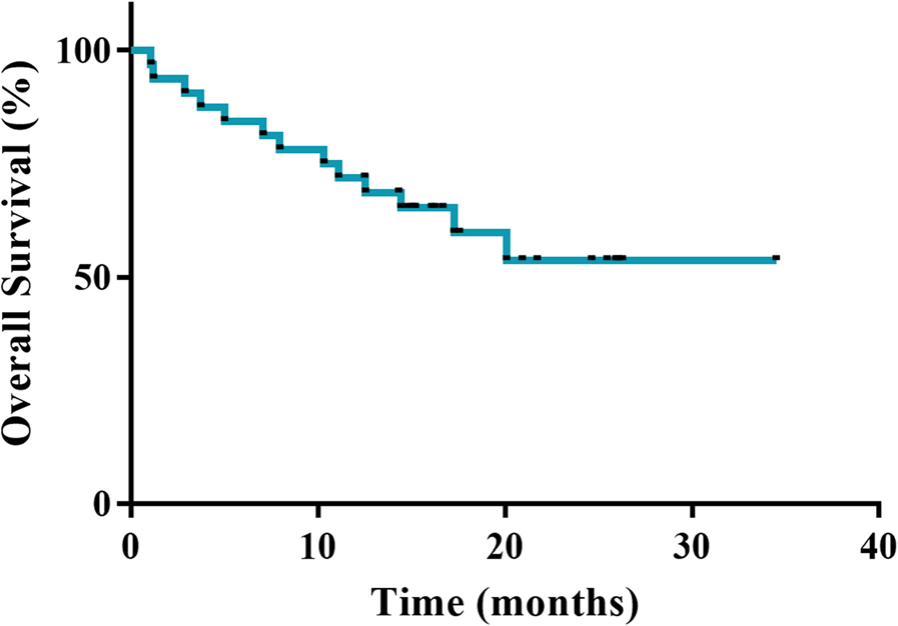
Kaplan-Meier Analysis of Overall Survival. The median OS was not reached as of May 2017. Abbreviation: OS = overall survival

**Fig. 5:**
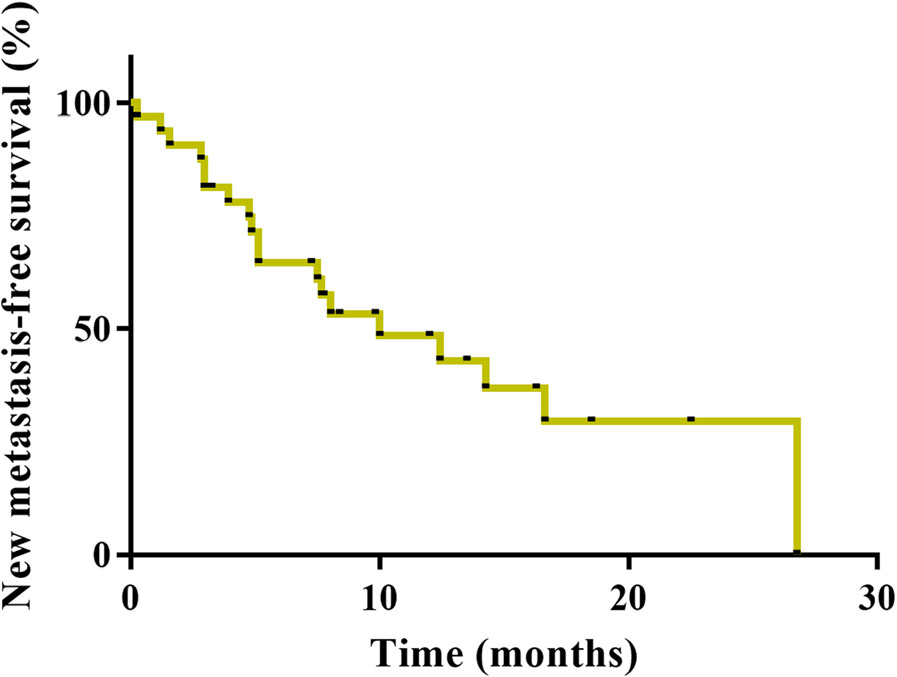
New Metastasis-free Survival. The new metastasis-free survival with experimental therapy was 9.2 months (95% CI: 5.1 to 14.8). New metastasis-free survival was defined as the time from randomization to death or disease progression due to a new metastasis

Of the 30 patients with measurable disease and sufficient repeat scans for assessing the primary endpoint of ORR (Table 2), the central review indicated CR in one (3.3%) and PR in nine (30%) patients. The ORR was 33.3% (95% CI: 17.3 to 52.8) and fell below the expected value of 35%. Nevertheless, the *P* value of the binomial test (one-sided exact test) for H0: 0.1 was 0.0005, thus the observed ORR was significantly higher than the threshold value of 10%. We also evaluated the response rate by metastatic organs (liver: 35.7%, lymph node: 10%, lung: 50%, skin: 100%). The DCR and CBR were 73.3% (*n* = 22; 95% CI: 54.1 to 87.7) and 43.3% (*n* = 13; 95% CI: 25.5 to 62.6), respectively. A waterfall plot analysis showed that most of the patients (83.3%) exhibited a decrease in the size of their tumours after receiving at least one dose of the study regimen (Fig. 2). As of May 2017, the median was 7.5 months (95% CI: 4.0 to 14.3) (Fig. 3). PFS results were based on a blinded independent central review. The median OS was not reached as of May 2017 (Fig. 4). The DOR was 7.8 months (95% CI: 1.9 to − 22.5) (data not shown). Disease progression due to new metastases occurred in nine patients (27.2%). An increase in the size of the pre-existing lesions occurred in five (15.2%) patients. The new metastasis-free survival after eribulin and S-1 combination therapy was 9.2 months (95% CI: 5.1 to 14.8) (Fig. 5).

**Table 2:** Tumour response

Response Category, n (%)	Eribulin/S-1 (*n* = 30)
Objective Response Rate; 95%CI	10 (33.3); 17.3–52.8
Complete Response	1 (3.3)
Partial Response	9 (30)
Stable Disease	12 (40)
Progressive Disease	7 (23.3)
Clinical Benefit Rate; 95%CI	13 (43.3); 25.5–62.6
Disease Control Rate; 95%CI	22 (73.3); 54.1–87.7

The ORR was 33.3% (95% CI: 17.3 to 52.8), the DCR was 73.3% (n = 22; 95% CI: 54.1 to 87.7), and the CBR was 43.3% (n = 13; 95% CI: 25.5–62.6)

In this study, ORR was evaluated only among patients who had measurable baseline disease according to the Response Evaluation Criteria in Solid Tumours, version 1.1

*Abbreviations*: *ORR* Overall response, *CBR* Clinical benefit rate: CR + PR + SD (> 6 months); *DCR* Disease control rate: CR + PR + SD

### Safety

The most commonly observed adverse events of any grade in the 32 patients who received at least one dose of the study drug were leukopenia (87.5%) and neutropenia (87.5%) (Table 3). The most commonly seen grade 3/4 haematological adverse events were neutropenia in 22 (68.8%) and leukopenia in 13 (40.6%) patients, both of which were clinically manageable. In contrast, grade 3/4 non-haematological toxicities included peripheral neuropathy in four (12.5%) patients and febrile neutropenia in three (9.4%) patients. Dose reductions were needed in 14 (43.7%) out of the 32 patients. There was only one death during the study attributed to disease progression, but there were no treatment-related deaths.

**Table 3:** Adverse events

AEs, n (%)	G1	G2	G3	G4	G3 + 4	All
Leukopenia	2 (6.3)	13 (40.6)	10 (31.3)	3 (9.4)	13 (40.6)	28 (87.5)
Neutropenia	1 (3.1)	5 (15.6)	8 (25)	14 (43.8)	22 (68.8)	28 (87.5)
Thrombocytopenia	14 (43.8)	2 (6.3)	1 (3.1)	0	1 (3.1)	17 (53.1)
Anemia	15 (46.9)	10 (31.3)	0	0	0	25 (78.1)
Febrile Neutropenia	0	0	3 (9.4)	0	3 (9.4)	3 (9.4)
Peripheral Neuropathy	0	9 (28.1)	4 (12.5)	0	4 (12.5)	13 (40.6)
Constipation	0	3 (9.4)	0	0	0	3 (9.4)
Dysgeusia	0	5 (15.6)	0	0	0	5 (15.6)
Fatigue	0	2 (6.3)	1 (3.1)	0	1 (3.1)	3 (9.4)
Fever	0	5 (15.6)	1 (3.1)	0	1 (3.1)	6 (18.8)
Oral Mucositis	0	4 (12.5)	1 (3.1)	0	1 (3.1)	5 (15.6)
Diarrhea	0	3 (9.4)	0	0	0	3 (9.4)

The most commonly seen grade 3/4 haematological adverse events were neutropenia in 22 (68.8%) and leukopenia in 13 (40.6%) patients. The grade 3/4 non-haematological toxicities included peripheral neuropathy in four (12.5%) patients and febrile neutropenia in three (9.4%) patients

## Discussion

CDK4/6 inhibitors are used in the treatment of hormone-positive and HER2-negative metastatic breast cancer patients as second line treatment are available worldwide [19]; however, this treatment is unsuitable for patients with rapid clinical progression and life-threatening visceral metastases. In this setting, chemotherapy is the standard therapy. However, there have only been a few clinical trials that have examined the efficacy and safety of the use of combination chemotherapies in patients with ABC whose prognoses were expected to be poor. Some of these clinical studies demonstrated that patients with ABC who were previously treated with anthracyclines had a worse prognosis and a lower chance of benefit from the anthracycline-containing regimens compared to those on non-anthracycline regimens [15]. Therefore, the development of alternative chemotherapies including combination therapy is warranted for this specific population. In our current study, we investigated the clinical activity and safety of eribulin in combination with S-1 in patients with ABC and a poor prognosis, as defined by the absence of hormone receptor expression, the presence of visceral involvement, presence of three or more metastatic sites, or shorter than 2 years of disease-free intervals. The ORR, which was the primary endpoint of the study, was 33.3%. Thus, this combination chemotherapy regimen might be of benefit for women with ABC and poor prognosis.

Patients with liver metastases from breast cancer have been shown to have a poor prognosis. Wyld et al. conducted a meta-analysis that included multiple chemotherapy regimens and reported that the median survival time in patients with hepatic metastases from breast cancer was only 4.23 months [20]. Fendler et al. also conducted a similar analysis and reported that the median survival time in 81 patients with hepatic metastases from breast cancer was 35 weeks [21]. Although our current study showed that the ORR among patients with liver metastasis (35.7%) was lower than those reported in the previous two studies (67.4 and 52%), we found that the OS in the present study (not reached but expected to be 10 months or longer) was longer than that in Wyld et al. (4.23 months, and 5.0 months) [20]. This suggests that the combination of eribulin and S-1 may have improved prognosis in this particular population.

Based on safety profiles of eribulin monotherapies [2, 22], grade 3 or 4 adverse events that occurred often with eribulin were neutropenia (45%), leucopenia (14%), and peripheral neuropathy (9%). In contrast, based on the safety profile of S-1 monotherapy, the most common grade 3 or worse adverse events were neutropenia (7%), fatigue (3%), and edema (< 1%) [11]. Results from combination therapy in MBC patients pre-treated with anthracycline and taxane [23, 24] showed that doublet agents produced significantly increased grade 3 or 4 leukopenia, anaemia, neutropenia, and gastrointestinal toxicities. Due to these toxicities, dose reduction and therapy discontinuation were necessary. In our study, the most commonly seen grade 3/4 adverse events were neutropenia (68.8%), leukopenia (40.6%), and peripheral sensory neuropathy (12.5%). The grade 3/4 haematological toxicities were increased compared to the respective monotherapies but gastrointestinal toxicities were not increased in our study. Although grade 3 peripheral sensory neuropathy was higher than the previous report, there were no patients with therapy discontinuation due to the toxicity in our study. In addition, most patients had an acceptable adverse event profile that made it possible for long-term administration without any dose reduction. Moreover, in the viewpoint of hepatic metastasis, 14 patients in our study had liver metastasis. Although these results are similar to the results of Wyld et al. [20], peripheral sensory neuropathy was higher in the present study. This might be attributable to the fact that there were more patients in our current study that had been previously treated with taxane (90%) compared to the previous study (76.3%). Therefore, a history of treatment with taxane may have led to the higher incidence of peripheral sensory neuropathy compared to the previous study. These results suggest that combination therapy is more toxic in terms of leukocytopenia and neutropenia, but tolerable for patients with poor prognosis. However, the sample size of the trials is small, and caution is advised when interpreting these results, since studies using a limited number of patients might have precluded infrequent treatment-emergent adverse events and overestimated the benefits in the population. Thus, further studies based on poor prognosis populations are urgently needed to confirm the results of our study.

Recently, combination chemotherapy showed a significant improvement in PFS and ORR in patients with MBC pre-treated with an anthracycline and a taxane, but did not benefit OS [23, 25]. Furthermore, meta-analysis demonstrated that combination therapy statistically improved OS, ORR and PFS in patients with HER2-negative ABC compared to sequential single agent therapy [24]. It has also been reported that the combination treatment of paclitaxel and gemcitabine resulted in a survival advantage in patients with ABC compared to paclitaxel alone [26]. Moreover, Park et al. reported that the efficacy of the combination treatment of eribulin and gemcitabine resulted in more favourable toxicity profiles compared to paclitaxel and gemcitabine [27]. Capecitabine in combination with docetaxel were additionally found to be superior to docetaxel alone in terms of OS and ORR in patients with ABC [28]. In a phase II study of S-1 monotherapy as second-line chemotherapy in MBC patients after resistance to anthracycline and taxane, S-1 demonstrated moderate efficacy with a PFS of 3.3 months and an ORR of 33.3% [29]. In a phase III study of S-1 as first-line chemotherapy for MBC, PFS was 9.6 months [11]. In contrast, a phase II study of eribulin monotherapy in MBC patients with anthracycline, taxane and capecitabine demonstrated that PFS was 2.6 months and ORR was 9.3% [22]. In our study, combination therapy demonstrated that PFS was 7.5 months and ORR was 33.3%, suggesting that our combination therapy improved PFS compared to each drug monotherapy. Furthermore, a phase II study of eribulin in combination with capecitabine has demonstrated that the ORR was 42.9% and the median PFS was 7.1 months (95% CI: 4.4 to 9.8) [30], compared to 33.3% and 7.5 months, respectively, in our current study. The lower ORR in the current versus the previous study was probably due to the fact that the current study focused on patients with a poor prognosis (hormone receptor-negative, presence of visceral involvement, presence of three or more metastatic sites, or a disease-free interval shorter than 2 years). When taken together, these results suggest that combination chemotherapy is the treatment of choice for patients with ABC, especially when there is a poor prognosis and good performance status.

Twelves et al. previously reported that patients with metastatic breast cancer who develop tumour progression with new metastases have a worse prognosis than patients whose disease progresses due to the growth of pre-existing lesions [31]. In a previous phase III study and EMBRACE study, new metastasis-free survival with eribulin monotherapy was 5.8 and 6.4 months, respectively [2, 3]. In our current study, new metastasis-free survival with eribulin and S-1 combination therapy was 9.2 months, which suggests that eribulin combined with S-1 improves the clinical outcome in ABC patients when compared to patients under eribulin monotherapy.

## Conclusion

Despite high incidence of grade 3 or 4 haematological toxicities, this phase II trial has demonstrated that use of combined treatment with eribulin and S-1 resulted in a high objective response rate, prolonged median PFS, and improved new metastasis-free survival with an acceptable safety profile. These results suggest that combined treatment with eribulin and S-1 is a potential option for patients with ABC and poor prognosis. Further studies are needed to validate the possible application of this treatment regime in practice.

## Data Availability

The datasets used and analysed during the current study are available from the corresponding author on reasonable request.

## Abbreviations

ABC: Advanced Breast cancer
CBR: Clinical benefit rate
CI: Confidence interval
CR: Complete response
CT: Combination chemotherapy
DCR: Disease control rate
DOR: Duration of response
HER2: Human Epidermal Growth Factor Receptor 2
ORR: Objective response rate
OS: Overall survival
PFS: Progression-free survival
RCTs: Randomised clinical trials
RECIST: Response Evaluation Criteria in Solid Tumors
SD: Stable disease
TTR: Time to recurrence
